# (*E*)-1-(1*H*-Benzotriazol-1-yl 3-oxide)-3-methoxy­but-2-en-1-one

**DOI:** 10.1107/S1600536809050442

**Published:** 2009-12-04

**Authors:** Jiu-Ming Li, Jian-Ping Yong

**Affiliations:** a College of Chemistry, Inner Mongolia University for Nationalities, Tongliao 028043, People’s Republic of China; b School of Public Health, Ningxia Medical University, Yinchuan 750004, People’s Republic of China

## Abstract

The title compound, C_11_H_11_N_3_O_3_, crystallizes with two independent mol­ecules of similar geometry in the asymmetric unit. The mol­ecular conformations are stabilized by intra­molecular C—H⋯O hydrogen bonds. The crystal packing consists of wave-like layers parallel to the *bc* plane formed by inter­molecular C—H⋯O hydrogen-bonding inter­actions involving only one independent mol­ecule.

## Related literature

For related structures, see: Barlos *et al.* (1985 ▶); Singh *et al.* (1988 ▶). For details of the biological activity of benzentriazol-containing compounds, see: Zhang *et al.* (2002 ▶). For comparative bond lengths, see: Allen *et al.* (1987 ▶).
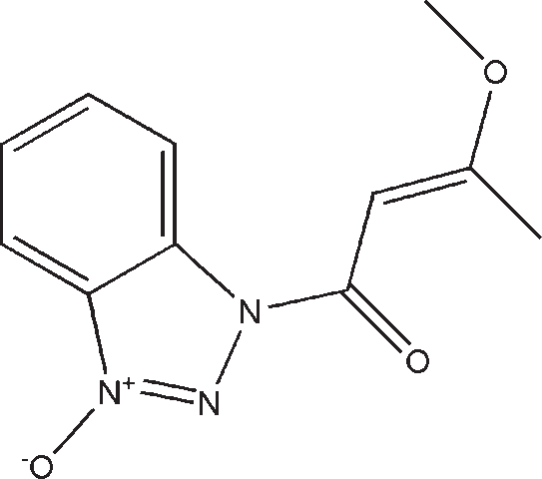


## Experimental

### 

#### Crystal data

C_11_H_11_N_3_O_3_
*M* *_r_* = 233.23
Monoclinic, 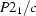

*a* = 14.011 (3) Å
*b* = 10.014 (2) Å
*c* = 15.699 (3) Å
β = 100.13 (3)°
*V* = 2168.3 (8) Å^3^
*Z* = 8
Mo *K*α radiation
μ = 0.11 mm^−1^
*T* = 173 K
0.40 × 0.40 × 0.30 mm

#### Data collection

Rigaku Mercury CCD/AFC diffractometer
Absorption correction: multi-scan (*CrystalClear*; Rigaku, 2007 ▶) *T*_min_ = 0.959, *T*_max_ = 0.969
15367 measured reflections
3771 independent reflections
3564 reflections with *I* > 2σ(*I*)
*R*_int_ = 0.050

#### Refinement

*R*[*F* ^2^ > 2σ(*F* ^2^)] = 0.063
*wR*(*F* ^2^) = 0.141
*S* = 1.17
3771 reflections
308 parameters
H-atom parameters constrained
Δρ_max_ = 0.36 e Å^−3^
Δρ_min_ = −0.28 e Å^−3^

### 

Data collection: *CrystalClear* (Rigaku, 2007 ▶); cell refinement: *CrystalClear*; data reduction: *CrystalClear*; program(s) used to solve structure: *SHELXS97* (Sheldrick, 2008 ▶); program(s) used to refine structure: *SHELXL97* (Sheldrick, 2008 ▶); molecular graphics: *SHELXTL* (Sheldrick, 2008 ▶); software used to prepare material for publication: *SHELXTL*.

## Supplementary Material

Crystal structure: contains datablocks I, global. DOI: 10.1107/S1600536809050442/rz2397sup1.cif

Structure factors: contains datablocks I. DOI: 10.1107/S1600536809050442/rz2397Isup2.hkl

Additional supplementary materials:  crystallographic information; 3D view; checkCIF report

## Figures and Tables

**Table 1:** Hydrogen-bond geometry (Å, °)

*D*—H⋯*A*	*D*—H	H⋯*A*	*D*⋯*A*	*D*—H⋯*A*
C2—H2*B*⋯O2	0.95	2.45	2.925 (3)	111
C13—H13*A*⋯O5	0.95	2.49	2.961 (3)	111
C14—H14*A*⋯O4^i^	0.95	2.57	3.398 (3)	147
C16—H16*A*⋯O4^ii^	0.95	2.45	3.379 (3)	165

Symmetry codes: (i) 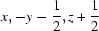; (ii) 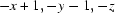.

**Fig. 1.**
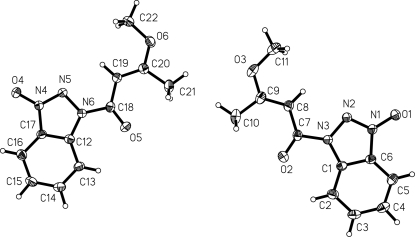
The molecular structure of the title compound, with atom-labelling scheme and 40% probability displacement ellipsoids.

## supplementary crystallographic information

### Comment

Benzotriazole derivatives exhibit good pharmacological activities and a wide spectrum of biological activities (Zhang *et al.*, 2002). In order to search for new benzotriazole compounds with higher bioactivity, we synthesized the title compound and describe its structure here.

The asymmetric unit of the title compound (Fig. 1) contains two independent molecules of similar geometry. The molecules are almost planar, the maximum deviation being 0.110 (2) Å for atom O2 in one molecule and 0.093 (3) Å for atom C22 in the other molecule. All bond lengths in the molecules are normal (Allen *et al.*, 1987) and in a good agreement with those reported previously for related compound (Barlos *et al.*, 1985; Singh *et al.*, 1988). The molecular conformations are stabilized by intramolecular C—H···O hydrogen bonds (Table 1). In the crystal packing, molecules containing the N4–N6 nitrogen atoms are linked by intermolecular C—H···O hydrogen bonds to form wavy layers parallel to the *bc* plane intersecting each other.

### Experimental

3-Methoxycrotonic acid (20 mmol) was dissolved in dichloromethane and cooled to 273 K, then 1-hydroxybenzotriazole (30 mmol) was added in one portion. After 10 h stirring at room temperature, the solution was washed successively with 1 N HCl and saturated NaCl, and the organic layer was separated, dried with Na_2_SO_4_ and evaporated to obtain the primary product. The pure compound was isolated by column chromatography (3.4 g, yield 73%). Single crystals suitable for X-ray measurements were obtained by slow evaporation of an ethyl acetate solution at room temperature. ^1^H NMR (400 MHz, CDCl3) δ: 8.49 (d, *J* = 8 Hz, 1H), 7.99 (d, *J* = 8 Hz, 1H), 7.75 (t, 1H), 7.53 (t, 1H), 6.30 (s, 1H), 3.85 (s, 3H), 2.50 (s, 3H).

### Refinement

H atoms were positioned geometrically and refined using a riding model, with C—H = 0.93–0.96 Å and with *U*_iso_(H) = 1.2 *U*_eq_(C) or 1.5 *U*_eq_(C) for methyl H atoms.

### Crystal data

**Table:**

C_11_H_11_N_3_O_3_	*F*(000) = 976
*M**_r_* = 233.23	*D*_x_ = 1.429 Mg m^−^^3^
Monoclinic, *P*2_1_/*c*	Mo *K*α radiation, λ = 0.71073 Å
Hall symbol: -P 2ybc	Cell parameters from 6607 reflections
*a* = 14.011 (3) Å	θ = 1.3–27.5°
*b* = 10.014 (2) Å	µ = 0.11 mm^−^^1^
*c* = 15.699 (3) Å	*T* = 173 K
β = 100.13 (3)°	Block, colourless
*V* = 2168.3 (8) Å^3^	0.40 × 0.40 × 0.30 mm
*Z* = 8	

### Data collection

**Table:**

Rigaku Mercury CCD/AFC diffractometer	3771 independent reflections
Radiation source: Sealed Tube	3564 reflections with *I* > 2σ(*I*)
Graphite Monochromator	*R*_int_ = 0.050
φ and ω scans	θ_max_ = 25.0°, θ_min_ = 1.5°
Absorption correction: multi-scan (*CrystalClear*; Rigaku, 2007)	*h* = −11→16
*T*_min_ = 0.959, *T*_max_ = 0.969	*k* = −11→11
15367 measured reflections	*l* = −18→18

### Refinement

**Table:**

Refinement on *F*^2^	Secondary atom site location: difference Fourier map
Least-squares matrix: full	Hydrogen site location: inferred from neighbouring sites
*R*[*F*^2^ > 2σ(*F*^2^)] = 0.063	H-atom parameters constrained
*wR*(*F*^2^) = 0.141	*w* = 1/[σ^2^(*F*_o_^2^) + (0.047*P*)^2^ + 1.4328*P*] where *P* = (*F*_o_^2^ + 2*F*_c_^2^)/3
*S* = 1.17	(Δ/σ)_max_ < 0.001
3771 reflections	Δρ_max_ = 0.36 e Å^−^^3^
308 parameters	Δρ_min_ = −0.28 e Å^−^^3^
0 restraints	Extinction correction: *SHELXL97* (Sheldrick, 2008), Fc^*^=kFc[1+0.001xFc^2^λ^3^/sin(2θ)]^-1/4^
Primary atom site location: structure-invariant direct methods	Extinction coefficient: 0.0028 (7)

### Special details

**Table:**

Geometry. All e.s.d.'s (except the e.s.d. in the dihedral angle between two l.s. planes) are estimated using the full covariance matrix. The cell e.s.d.'s are taken into account individually in the estimation of e.s.d.'s in distances, angles and torsion angles; correlations between e.s.d.'s in cell parameters are only used when they are defined by crystal symmetry. An approximate (isotropic) treatment of cell e.s.d.'s is used for estimating e.s.d.'s involving l.s. planes.
Refinement. Refinement of *F*^2^ against ALL reflections. The weighted *R*-factor *wR* and goodness of fit *S* are based on *F*^2^, conventional *R*-factors *R* are based on *F*, with *F* set to zero for negative *F*^2^. The threshold expression of *F*^2^ > σ(*F*^2^) is used only for calculating *R*-factors(gt) *etc*. and is not relevant to the choice of reflections for refinement. *R*-factors based on *F*^2^ are statistically about twice as large as those based on *F*, and *R*- factors based on ALL data will be even larger.

### Fractional atomic coordinates and isotropic or equivalent isotropic displacement parameters (Å^2^)

**Table:**

	*x*	*y*	*z*	*U*_iso_*/*U*_eq_	
O1	0.05575 (14)	1.36306 (16)	−0.08605 (11)	0.0400 (5)	
O2	0.14314 (14)	0.85371 (17)	0.05299 (11)	0.0411 (5)	
O3	0.18500 (13)	0.72121 (18)	−0.19749 (12)	0.0436 (5)	
O4	0.45305 (13)	−0.36283 (16)	−0.08904 (11)	0.0387 (4)	
O5	0.33528 (13)	0.14842 (17)	−0.00327 (10)	0.0383 (4)	
O6	0.32908 (13)	0.26568 (17)	−0.26560 (11)	0.0380 (4)	
N1	0.07616 (14)	1.25933 (19)	−0.03951 (12)	0.0297 (5)	
N2	0.09586 (14)	1.14406 (18)	−0.07177 (12)	0.0292 (4)	
N3	0.11216 (14)	1.05828 (19)	−0.00260 (11)	0.0272 (4)	
N4	0.43065 (14)	−0.25590 (19)	−0.05301 (12)	0.0282 (4)	
N5	0.40993 (14)	−0.14465 (19)	−0.09592 (12)	0.0286 (4)	
N6	0.38814 (13)	−0.05422 (19)	−0.03598 (11)	0.0264 (4)	
C1	0.10201 (16)	1.1226 (2)	0.07363 (14)	0.0278 (5)	
C2	0.10832 (17)	1.0824 (3)	0.15936 (15)	0.0341 (6)	
H2B	0.1237	0.9932	0.1773	0.041*	
C3	0.09079 (18)	1.1801 (3)	0.21672 (15)	0.0375 (6)	
H3A	0.0940	1.1566	0.2758	0.045*	
C4	0.06852 (18)	1.3119 (3)	0.19175 (16)	0.0377 (6)	
H4A	0.0574	1.3748	0.2343	0.045*	
C5	0.06218 (17)	1.3534 (2)	0.10725 (16)	0.0337 (6)	
H5B	0.0473	1.4428	0.0895	0.040*	
C6	0.07931 (16)	1.2541 (2)	0.05007 (14)	0.0280 (5)	
C7	0.13675 (16)	0.9212 (2)	−0.01186 (15)	0.0286 (5)	
C8	0.15008 (16)	0.8828 (2)	−0.09752 (15)	0.0302 (5)	
H8A	0.1417	0.9484	−0.1419	0.036*	
C9	0.17412 (17)	0.7568 (2)	−0.11732 (16)	0.0336 (6)	
C10	0.1935 (2)	0.6417 (3)	−0.0578 (2)	0.0485 (7)	
H10A	0.2091	0.5631	−0.0900	0.073*	
H10B	0.1358	0.6232	−0.0323	0.073*	
H10C	0.2482	0.6625	−0.0118	0.073*	
C11	0.1690 (2)	0.8191 (3)	−0.26569 (18)	0.0473 (7)	
H11A	0.1797	0.7781	−0.3199	0.071*	
H11B	0.2143	0.8937	−0.2509	0.071*	
H11C	0.1023	0.8522	−0.2726	0.071*	
C12	0.39549 (16)	−0.1127 (2)	0.04485 (14)	0.0271 (5)	
C13	0.38077 (17)	−0.0679 (3)	0.12604 (15)	0.0326 (6)	
H13A	0.3613	0.0211	0.1350	0.039*	
C14	0.39604 (18)	−0.1599 (3)	0.19193 (16)	0.0368 (6)	
H14A	0.3867	−0.1329	0.2479	0.044*	
C15	0.42483 (18)	−0.2921 (3)	0.18018 (16)	0.0367 (6)	
H15A	0.4350	−0.3513	0.2282	0.044*	
C16	0.43866 (17)	−0.3378 (2)	0.10031 (15)	0.0333 (6)	
H16A	0.4572	−0.4271	0.0911	0.040*	
C17	0.42345 (16)	−0.2437 (2)	0.03457 (14)	0.0273 (5)	
C18	0.35556 (16)	0.0780 (2)	−0.06071 (15)	0.0293 (5)	
C19	0.35254 (16)	0.1095 (2)	−0.15081 (15)	0.0289 (5)	
H19A	0.3658	0.0412	−0.1891	0.035*	
C20	0.33127 (17)	0.2343 (2)	−0.18219 (15)	0.0307 (5)	
C21	0.3085 (2)	0.3529 (2)	−0.13222 (18)	0.0404 (6)	
H21A	0.2960	0.4300	−0.1710	0.061*	
H21B	0.3637	0.3723	−0.0862	0.061*	
H21C	0.2510	0.3344	−0.1066	0.061*	
C22	0.3452 (2)	0.1631 (3)	−0.32498 (16)	0.0415 (6)	
H22A	0.3413	0.2015	−0.3829	0.062*	
H22B	0.2957	0.0936	−0.3265	0.062*	
H22C	0.4096	0.1240	−0.3062	0.062*	

### Atomic displacement parameters (Å^2^)

**Table:**

	*U*^11^	*U*^22^	*U*^33^	*U*^12^	*U*^13^	*U*^23^
O1	0.0628 (12)	0.0248 (9)	0.0349 (9)	0.0044 (8)	0.0158 (9)	0.0052 (8)
O2	0.0557 (12)	0.0323 (10)	0.0359 (10)	0.0058 (8)	0.0098 (8)	0.0062 (8)
O3	0.0470 (11)	0.0364 (10)	0.0493 (11)	−0.0006 (8)	0.0138 (9)	−0.0164 (9)
O4	0.0562 (11)	0.0268 (9)	0.0349 (9)	0.0079 (8)	0.0128 (8)	−0.0030 (7)
O5	0.0519 (11)	0.0336 (9)	0.0302 (9)	0.0057 (8)	0.0090 (8)	−0.0069 (8)
O6	0.0448 (10)	0.0327 (10)	0.0388 (10)	0.0074 (8)	0.0132 (8)	0.0094 (8)
N1	0.0380 (11)	0.0234 (10)	0.0289 (10)	−0.0006 (8)	0.0090 (9)	−0.0008 (8)
N2	0.0380 (11)	0.0235 (10)	0.0266 (10)	−0.0004 (8)	0.0075 (8)	0.0001 (8)
N3	0.0335 (11)	0.0257 (10)	0.0231 (9)	0.0003 (8)	0.0066 (8)	0.0001 (8)
N4	0.0330 (11)	0.0247 (10)	0.0279 (10)	0.0001 (8)	0.0082 (8)	0.0003 (8)
N5	0.0352 (11)	0.0260 (10)	0.0255 (10)	0.0011 (8)	0.0078 (8)	−0.0011 (8)
N6	0.0306 (10)	0.0257 (10)	0.0232 (9)	−0.0003 (8)	0.0054 (8)	−0.0001 (8)
C1	0.0260 (12)	0.0314 (13)	0.0264 (12)	−0.0019 (10)	0.0057 (9)	−0.0021 (10)
C2	0.0334 (13)	0.0417 (14)	0.0271 (12)	0.0033 (11)	0.0050 (10)	0.0032 (11)
C3	0.0367 (14)	0.0526 (16)	0.0235 (12)	−0.0021 (12)	0.0063 (10)	−0.0025 (11)
C4	0.0358 (13)	0.0477 (16)	0.0301 (13)	−0.0028 (12)	0.0073 (11)	−0.0125 (12)
C5	0.0331 (13)	0.0307 (13)	0.0376 (13)	−0.0031 (10)	0.0068 (11)	−0.0073 (11)
C6	0.0310 (12)	0.0288 (12)	0.0246 (11)	−0.0039 (10)	0.0056 (10)	−0.0026 (10)
C7	0.0272 (12)	0.0248 (12)	0.0341 (13)	−0.0010 (9)	0.0066 (10)	0.0006 (10)
C8	0.0305 (12)	0.0269 (12)	0.0334 (13)	−0.0014 (10)	0.0064 (10)	−0.0017 (10)
C9	0.0276 (12)	0.0302 (13)	0.0433 (14)	−0.0027 (10)	0.0073 (11)	−0.0075 (11)
C10	0.0504 (17)	0.0285 (14)	0.0657 (19)	0.0052 (12)	0.0080 (15)	−0.0031 (13)
C11	0.0534 (17)	0.0480 (17)	0.0427 (15)	−0.0070 (14)	0.0149 (13)	−0.0159 (13)
C12	0.0250 (11)	0.0314 (13)	0.0243 (11)	−0.0047 (9)	0.0029 (9)	0.0002 (10)
C13	0.0374 (13)	0.0348 (13)	0.0265 (12)	−0.0012 (11)	0.0085 (10)	−0.0040 (10)
C14	0.0387 (14)	0.0459 (15)	0.0267 (12)	−0.0043 (12)	0.0084 (11)	−0.0023 (11)
C15	0.0402 (14)	0.0419 (15)	0.0285 (12)	−0.0060 (12)	0.0073 (11)	0.0068 (11)
C16	0.0334 (13)	0.0321 (13)	0.0343 (13)	−0.0046 (10)	0.0060 (11)	0.0032 (11)
C17	0.0290 (12)	0.0296 (12)	0.0235 (11)	−0.0043 (9)	0.0049 (9)	−0.0016 (9)
C18	0.0281 (12)	0.0259 (12)	0.0332 (13)	−0.0009 (9)	0.0035 (10)	−0.0028 (10)
C19	0.0313 (12)	0.0269 (12)	0.0285 (12)	−0.0010 (10)	0.0047 (10)	−0.0013 (10)
C20	0.0277 (12)	0.0312 (13)	0.0344 (13)	0.0002 (10)	0.0085 (10)	0.0023 (10)
C21	0.0433 (15)	0.0274 (13)	0.0523 (16)	0.0035 (11)	0.0131 (13)	0.0013 (12)
C22	0.0553 (17)	0.0390 (15)	0.0317 (13)	0.0053 (12)	0.0118 (12)	0.0062 (11)

### Geometric parameters (Å, °)

**Table:**

O1—N1	1.273 (2)	C8—C9	1.356 (3)
O2—C7	1.212 (3)	C8—H8A	0.9500
O3—C9	1.342 (3)	C9—C10	1.478 (4)
O3—C11	1.440 (3)	C10—H10A	0.9800
O4—N4	1.276 (2)	C10—H10B	0.9800
O5—C18	1.217 (3)	C10—H10C	0.9800
O6—C20	1.342 (3)	C11—H11A	0.9800
O6—C22	1.431 (3)	C11—H11B	0.9800
N1—N2	1.309 (3)	C11—H11C	0.9800
N1—C6	1.400 (3)	C12—C17	1.386 (3)
N2—N3	1.372 (3)	C12—C13	1.400 (3)
N3—C1	1.388 (3)	C13—C14	1.373 (3)
N3—C7	1.429 (3)	C13—H13A	0.9500
N4—N5	1.308 (3)	C14—C15	1.406 (4)
N4—C17	1.401 (3)	C14—H14A	0.9500
N5—N6	1.378 (3)	C15—C16	1.380 (3)
N6—C12	1.385 (3)	C15—H15A	0.9500
N6—C18	1.432 (3)	C16—C17	1.385 (3)
C1—C6	1.389 (3)	C16—H16A	0.9500
C1—C2	1.393 (3)	C18—C19	1.442 (3)
C2—C3	1.381 (4)	C19—C20	1.357 (3)
C2—H2B	0.9500	C19—H19A	0.9500
C3—C4	1.396 (4)	C20—C21	1.488 (3)
C3—H3A	0.9500	C21—H21A	0.9800
C4—C5	1.378 (4)	C21—H21B	0.9800
C4—H4A	0.9500	C21—H21C	0.9800
C5—C6	1.389 (3)	C22—H22A	0.9800
C5—H5B	0.9500	C22—H22B	0.9800
C7—C8	1.442 (3)	C22—H22C	0.9800
			
C9—O3—C11	119.2 (2)	H10A—C10—H10C	109.5
C20—O6—C22	119.25 (19)	H10B—C10—H10C	109.5
O1—N1—N2	122.60 (18)	O3—C11—H11A	109.5
O1—N1—C6	124.83 (19)	O3—C11—H11B	109.5
N2—N1—C6	112.57 (18)	H11A—C11—H11B	109.5
N1—N2—N3	105.25 (17)	O3—C11—H11C	109.5
N2—N3—C1	111.34 (18)	H11A—C11—H11C	109.5
N2—N3—C7	122.05 (18)	H11B—C11—H11C	109.5
C1—N3—C7	126.62 (19)	N6—C12—C17	105.70 (19)
O4—N4—N5	122.44 (18)	N6—C12—C13	134.4 (2)
O4—N4—C17	125.01 (19)	C17—C12—C13	119.9 (2)
N5—N4—C17	112.55 (18)	C14—C13—C12	116.2 (2)
N4—N5—N6	105.29 (16)	C14—C13—H13A	121.9
N5—N6—C12	110.97 (18)	C12—C13—H13A	121.9
N5—N6—C18	121.38 (18)	C13—C14—C15	122.9 (2)
C12—N6—C18	127.49 (19)	C13—C14—H14A	118.6
N3—C1—C6	105.28 (19)	C15—C14—H14A	118.6
N3—C1—C2	134.4 (2)	C16—C15—C14	121.4 (2)
C6—C1—C2	120.3 (2)	C16—C15—H15A	119.3
C3—C2—C1	115.9 (2)	C14—C15—H15A	119.3
C3—C2—H2B	122.0	C15—C16—C17	115.1 (2)
C1—C2—H2B	122.0	C15—C16—H16A	122.5
C2—C3—C4	122.9 (2)	C17—C16—H16A	122.5
C2—C3—H3A	118.5	C16—C17—C12	124.5 (2)
C4—C3—H3A	118.5	C16—C17—N4	130.1 (2)
C5—C4—C3	121.9 (2)	C12—C17—N4	105.49 (19)
C5—C4—H4A	119.0	O5—C18—N6	116.1 (2)
C3—C4—H4A	119.0	O5—C18—C19	129.0 (2)
C4—C5—C6	114.7 (2)	N6—C18—C19	114.9 (2)
C4—C5—H5B	122.7	C20—C19—C18	121.6 (2)
C6—C5—H5B	122.7	C20—C19—H19A	119.2
C5—C6—C1	124.3 (2)	C18—C19—H19A	119.2
C5—C6—N1	130.1 (2)	O6—C20—C19	122.4 (2)
C1—C6—N1	105.56 (19)	O6—C20—C21	111.0 (2)
O2—C7—N3	115.7 (2)	C19—C20—C21	126.7 (2)
O2—C7—C8	129.2 (2)	C20—C21—H21A	109.5
N3—C7—C8	115.1 (2)	C20—C21—H21B	109.5
C9—C8—C7	122.7 (2)	H21A—C21—H21B	109.5
C9—C8—H8A	118.7	C20—C21—H21C	109.5
C7—C8—H8A	118.7	H21A—C21—H21C	109.5
O3—C9—C8	122.4 (2)	H21B—C21—H21C	109.5
O3—C9—C10	110.3 (2)	O6—C22—H22A	109.5
C8—C9—C10	127.3 (2)	O6—C22—H22B	109.5
C9—C10—H10A	109.5	H22A—C22—H22B	109.5
C9—C10—H10B	109.5	O6—C22—H22C	109.5
H10A—C10—H10B	109.5	H22A—C22—H22C	109.5
C9—C10—H10C	109.5	H22B—C22—H22C	109.5
			
O1—N1—N2—N3	−179.03 (19)	C11—O3—C9—C8	−1.1 (3)
C6—N1—N2—N3	0.4 (2)	C11—O3—C9—C10	179.8 (2)
N1—N2—N3—C1	0.1 (2)	C7—C8—C9—O3	179.0 (2)
N1—N2—N3—C7	−179.59 (19)	C7—C8—C9—C10	−2.1 (4)
O4—N4—N5—N6	178.92 (19)	N5—N6—C12—C17	0.4 (2)
C17—N4—N5—N6	0.1 (2)	C18—N6—C12—C17	175.7 (2)
N4—N5—N6—C12	−0.3 (2)	N5—N6—C12—C13	−179.2 (2)
N4—N5—N6—C18	−175.93 (19)	C18—N6—C12—C13	−3.9 (4)
N2—N3—C1—C6	−0.5 (2)	N6—C12—C13—C14	179.9 (2)
C7—N3—C1—C6	179.1 (2)	C17—C12—C13—C14	0.3 (3)
N2—N3—C1—C2	178.3 (2)	C12—C13—C14—C15	0.0 (4)
C7—N3—C1—C2	−2.0 (4)	C13—C14—C15—C16	−0.6 (4)
N3—C1—C2—C3	−178.7 (2)	C14—C15—C16—C17	0.9 (3)
C6—C1—C2—C3	0.0 (3)	C15—C16—C17—C12	−0.6 (3)
C1—C2—C3—C4	−0.4 (4)	C15—C16—C17—N4	−179.8 (2)
C2—C3—C4—C5	0.3 (4)	N6—C12—C17—C16	−179.7 (2)
C3—C4—C5—C6	0.2 (4)	C13—C12—C17—C16	0.0 (4)
C4—C5—C6—C1	−0.5 (4)	N6—C12—C17—N4	−0.3 (2)
C4—C5—C6—N1	177.9 (2)	C13—C12—C17—N4	179.4 (2)
N3—C1—C6—C5	179.5 (2)	O4—N4—C17—C16	0.7 (4)
C2—C1—C6—C5	0.4 (4)	N5—N4—C17—C16	179.5 (2)
N3—C1—C6—N1	0.7 (2)	O4—N4—C17—C12	−178.6 (2)
C2—C1—C6—N1	−178.3 (2)	N5—N4—C17—C12	0.2 (3)
O1—N1—C6—C5	0.0 (4)	N5—N6—C18—O5	177.7 (2)
N2—N1—C6—C5	−179.4 (2)	C12—N6—C18—O5	2.8 (3)
O1—N1—C6—C1	178.7 (2)	N5—N6—C18—C19	−3.0 (3)
N2—N1—C6—C1	−0.7 (3)	C12—N6—C18—C19	−177.9 (2)
N2—N3—C7—O2	−175.4 (2)	O5—C18—C19—C20	5.0 (4)
C1—N3—C7—O2	5.0 (3)	N6—C18—C19—C20	−174.2 (2)
N2—N3—C7—C8	4.4 (3)	C22—O6—C20—C19	3.0 (3)
C1—N3—C7—C8	−175.3 (2)	C22—O6—C20—C21	−177.4 (2)
O2—C7—C8—C9	−0.7 (4)	C18—C19—C20—O6	179.3 (2)
N3—C7—C8—C9	179.6 (2)	C18—C19—C20—C21	−0.2 (4)

### Hydrogen-bond geometry (Å, °)

**Table:**

*D*—H···*A*	*D*—H	H···*A*	*D*···*A*	*D*—H···*A*
C2—H2B···O2	0.95	2.45	2.925 (3)	111
C13—H13A···O5	0.95	2.49	2.961 (3)	111
C14—H14A···O4^i^	0.95	2.57	3.398 (3)	147
C16—H16A···O4^ii^	0.95	2.45	3.379 (3)	165

Symmetry codes: (i) *x*, −*y*−1/2, *z*+1/2; (ii) −*x*+1, −*y*−1, −*z*.
